# Distinct gene expression patterns in vector-residing *Leishmania infantum* identify parasite stage-enriched markers

**DOI:** 10.1371/journal.pntd.0008014

**Published:** 2020-03-03

**Authors:** Iliano V. Coutinho-Abreu, Tiago D. Serafim, Claudio Meneses, Shaden Kamhawi, Fabiano Oliveira, Jesus G. Valenzuela

**Affiliations:** Vector Molecular Biology Section, Laboratory of Malaria and Vector Research, National Institute of Allergy and Infectious Diseases, National Institutes of Health, Rockville, Maryland, United States of America; Lancaster University, UNITED KINGDOM

## Abstract

**Background:**

Leishmaniasis is a vector-borne neglected disease. Inside the natural sand fly vector, the promastigote forms of *Leishmania* undergo a series of extracellular developmental stages to reach the infectious stage, the metacyclic promastigote. There is limited information regarding the expression profile of *L*. *infantum* developmental stages inside the sand fly vector, and molecular markers that can distinguish the different parasite stages are lacking.

**Methodology/Principal findings:**

We performed RNAseq on unaltered midguts of the sand fly *Lutzomyia longipalpis* after infection with *L*. *infantum* parasites. RNAseq was carried out at various time points throughout parasite development. Principal component analysis separated the transcripts corresponding to the different *Leishmania* promastigote stages, the procyclic, nectomonad, leptomonad and metacyclics. Importantly, there were a significant number of differentially expressed genes when comparing the sequential development of the various *Leishmania* stages in the sand fly. There were 836 differentially expressed (DE) genes between procyclic and long nectomonad promastigotes; 113 DE genes between nectomonad and leptomonad promastigotes; and 302 DE genes between leptomonad and metacyclic promastigotes. Most of the DE genes do not overlap across stages, highlighting the uniqueness of each *Leishmania* stage. Furthermore, the different stages of *Leishmania* parasites exhibited specific transcriptional enrichment across chromosomes. Using the transcriptional signatures exhibited by distinct *Leishmania* stages during their development in the sand fly midgut, we determined the genes predominantly enriched in each stage, identifying multiple potential stage-specific markers for *L*. *infantum*.

**Conclusions:**

Overall, these findings demonstrate the transcriptional plasticity of the *Leishmania* parasite inside the sand fly vector and provide a repertoire of potential stage-specific markers for further development as molecular tools for epidemiological studies.

## Author summary

One of the major gaps in *Leishmania* research is the lack of stage-specific markers to differentiate the *Leishmania* promastigote stages in the sand fly vector as well as in culture. In this study, we report the transcriptional boundaries amongst *Leishmania infantum* promastigote stages developing in the sand fly vector *Lutzomyia longipalpis*. Despite the policystronic nature of *Leishmania* mRNAs, we were able to detect 10- to 100 fold differences in transcriptional abundance between genes throught RNA sequencing. Significant number of genes were differentially expressed between subsequent stages, showing enrichment of stage specific up-regulated genes even across chromosomes. In light of such differences, we identified potential stage-specific markers for each promastigote stage of *Leishmania* with potential use in epidemiological studies as well as in further characterization the developmental differences amongst the promastigote stages in the vector.

## Introduction

*Leishmania* parasites are diploid single cell organisms, bearing between 34–36 chromosomes [1]. In clinical isolates, the *Leishmania* karyotype is very plastic, with striking differences not only between geographic isolates [2], but also in parasites isolated from different organs of identical patients [3, 4]. Differences in aneuploidy but also gene copy number variation (CNV) account for most of gene expression variations between *Leishmania* strains or clinical isolates [2, 5]. These differences are associated with *Leishmania* virulence and drug resistance [3, 6], likely representing an evolutionary adaptation for growth in the sand fly vector and human host [3, 4, 6]. Surprisingly, < 70 species-specific genes have been found among *Leishmania* species [1].

*Leishmania* are digenetic parasites, switching between mammalian hosts and sand fly vectors. When taken up in a sand fly blood meal, the amastigote stage of *Leishmania* enlarges and exposes the flagellum, undergoing differentiation to the procyclic stage (0.3 fold flagellum to body length ratio) [7]. This transformation is followed by multiple rounds of cell division inside the insect gut within the confinement of a newly synthesized peritrophic matrix (PM). As the sand fly PM matures, *Leishmania* procyclics elongate their cell bodies to twice the procyclic size, giving rise to the nectomonad stage (0.9 fold flagellum to body length ratio) [7]. Upon breakdown of the PM, the nectomonads escape to the midgut lumen, with some parasites migrating straight to the cardia where they differentiate into haptomonads and eventually form a haptomonad parasite sphere [7, 8]. The free swimming nectomonads attach to the midgut epithelium and give rise to a form displaying a longer flagellum (1.2–1.9 fold flagellum to body length ratio), the leptomonad [9]. Leptomonads undergo multiple rounds of division and move anteriorly along the thoracic midgut as they secrete high amounts of filamentous proteophosphoglycan forming a secretory gel (PSG) [9]. Afterwards, leptomonads begin to shrink their cell bodies and elongate their flagellum, giving rise to the infective forms, the metacyclic parasites. As the infection matures, the proportion of metacyclics relative to the other stages increases with time reaching as high as 80–90% [7, 8, 10, 11].

Similar to other Trypanosomatids, *Leishmania* genes are transcribed as long polycistronic RNAs by RNA polymerase II [12, 13]. Such long RNAs are subsequently processed by trans splicing: addition of a capped splice leader sequence at the 5’ end followed up by cleavage and polyadenylation at the 3’ end of each protein-coding unit [12]. Initial microarray studies have identified a low differential expression (< 5% DE genes) between two *Leishmania* life stages (amastigotes and promastigotes from culture), highlighting a disconnect between transcription and translation. These findings suggest that the *Leishmania* genome is constitutively transcribed, and the control of gene expression is carried out post-transcriptionally at the level of RNA processing and/or translation [13]. Conversely, high throughput RNA sequencing of *Leishmania* transcriptomes detected gene expression differences between intracellular (human host) and extracellular (vector host) parasite stages [14]. Comparing *L*. *major* culture promastigotes and murine macrophage amastigotes, or culture promastigotes and human macrophage amastigotes, at least 30% of the genes were differentially expressed (q-value <0.05) [15–17].

Apart from a recent work investigating *L*. *major* stages inside the *Phlebotomus papatasi* sand fly [18], and comparison between culture- and *Ph*. *perniciosus*- derived *L*. *infantum* late stage parasites [19], little is known of molecular markers and RNA differential expression between the *Leishmania* promastigote stages developing in the midgut of the sand fly vector, reviewed in [20], particularly for *L*. *infantum* inside its natural sand fly vector, *Lutzomyia longipalpis*. In order to fill this knowledge gap, we performed a comprehensive RNAseq investigation to assess *L*. *infantum* gene expression in the midgut of *Lu*. *longipalpis* at six time points corresponding to each developmental stage, from procyclic to infective metacyclic promastigotes. Lastly, we identified candidate genes as stage-specific markers for *L*. *infantum* that will provide a valuable tool for characterizing *Leishmania* stages in the sand fly vector.

## Methods

### Ethics statement

All animal experimental procedures were reviewed and approved by the National Institute of Allergy and Infectious Diseases (NIAID) Animal Care and Use Committee under animal protocol LMVR4E. The NIAID DIR Animal Care and Use Program complies with the Guide for the Care and Use of Laboratory Animals and with the NIH Office of Animal Care and Use and Animal Research Advisory Committee guidelines. Detailed NIH Animal Research Guidelines can be accessed at https://oma1.od.nih.gov/manualchapters/intramural/3040-2/.

### *Leishmania* parasites, sand fly blood feeding and infection, and midgut dissection and storage

The strain of *L*. *infantum* (MCAN/BR/09/52) used in this study was isolated from a spleen of a dog from Natal, Brazil [21]. The amastigotes used for the sand fly infections were harvested from the spleens of Golden Syrian hamsters, as previously described [22]. Frozen amastigotes were washed once in 1X PBS and five million parasites were inoculated into 1mL of heparinized dog blood. Dog blood was provided by the Division of Veterinary Research at the National Institutes of Health. *Leishmania*-seeded blood was loaded into a custom-made glass feeder (Chemglass Life Science, CG183570), capped with a chick skin. The glass feeder was kept at 37°C by circulating heated water. The sand fly *L*. *longipalpis* was allowed to feed for three hours in the dark. As controls, *L*. *longipalpis* sand flies were also fed on uninfected heparinized dog blood at the same time. After feeding, fully fed females were sorted and given 30% sucrose solution *ad libitum*. Sand flies from both groups were dissected with fine needles and tweezers on a glass slide at days two, four, six, eight, twelve, and fourteen after blood feeding on RNAse Free PBS (1X). Forty to sixty midguts were quickly rinsed in fresh RNAse Free PBS (1X) and stored in RNAlater (Ambion), following manufacturer’s recommendation. We then performed RNAseq on RNA of *Leishmania*-infected sand fly midguts to prevent potential bias in gene expression that can be generated by purifying *Leishmania* before RNAseq [14]. Experiments were were carried out in three biological replicates.

### Parasite load assessment

A few infected sand fly midguts from all dissected time points were also used to measure parasite loads using Neubauer improved chamber (Incyto, DNC-NO1), as described by the manufacturer. Briefly, dissected midguts were individually transferred to 1.7 mL microtubes (Denville Scientific, C2172) containing 30 μL of 1X PBS and homogenized with a disposable pellet mixer and a cordless motor (Kimble, 7495400000). In order to count the fast moving metacyclic stage parasites, formalin was added to the PBS solution to a final concentration of 0.005%. The Neubauer chamber was loaded with 10 μL of the midgut homogenate (or dilutions of such), and parasites were counted under a microscope (Axiostar plus, Zeiss) at 400X magnification. As the parasite loads on day two are very low and the parasites difficult to be detected due to the blood remains, parasites were not counted at this time point.

### RNA extraction and quality control

Total RNA was extracted using the PureLink RNA Mini Kit (Life Technologies, Carlsbad), following the manufacturer’s recommendations. Briefly, excess RNA later was removed by pipetting, and sample homogenization on lysis buffer was also carried out by pipetting samples up and down for about 60 times. Each sample was eluted into 35μL of RNAse free water.

Sample concentration was measured by Nanodrop spectrophotometer (Nano Drop Technologies Inc, Wilmingtom; ND-1000), and RNA quality was assessed by Bioanalyzer (Agilent Technologies Inc, Santa Clara, CA; 2100 Bioanalyzer), using the Agilent RNA 6000 Nano kit (Agilent Technologies) and following the manufacturer’s recommendations. Only one out of the forty eight samples displayed RIN (RNA integrity number) value lower than 7 (Replicate 3–14d Pi–RIN 6.7).

### RNAseq library preparation and deep sequencing

The RNASeq library preparation and sequencing was performed at the NC State University Genomic Science Laboratory. The RNAseq libraries were constructed using the NEBNext Ultra RNA Library Prep Kit for Illumina (New England Biolabs, Ipswick MA), following manufacture’s recommendation, in order to obtain reads of 125 nucleotides. RNA libraries were sequenced (Single Ended– 125 SE) in three lanes of the HiSeq 2500 (Illumina, San Diego, CA).

### RNA-seq data trimming, mapping, and differential expression analysis

Raw RNA sequences were trimmed with trimmomatic vs. 0.36 [23] in order to remove poor quality sequences and adaptors using parameters: ILLUMINACLIP:2:30:10 LEADING:3 TRAILING:3 SLIDINGWINDOW:4:15 MINLEN:36. After trimming, quality control of FASTQ sequences were assessed with the FASTQC software (Babraham Bioinfomatics, http://www.bioinformatics.babraham.ac.uk/projects/fastqc/). Trimmed reads were then mapped and counts estimated against the *L*. *infantum* JPCM5 genome (assembly ASM287v2) using the RNA-Seq by Expectation Maximization (RSEM) vs 1.3.0, Bowtie vs 2–2.2.5 and samtools vs 1.2 [24]. Differential expression among timepoints and conditions were analyzed using the R suite by the Bioconductor package DeSeq2 vs 3.8 [25]. Filtering on all mapped gene counts was performed to exclude genes where the sum of counts in all the conditions was inferior to 10 counts. Default parameters were used with DESeq2 including the shrinks log_2_ fold-change (FC) estimated for each tested comparison [25, 26]. A log_2_ FoldChange and its standard error were generated in addition to a P-value (p value) and a P-adj (Adjusted p-value) to account for the false discovery rate. Significant associations were considered when a P-adj was smaller than 5% (p <0.05) and log_2_ fold change larger than 0.5 (+/-). To classify families of genes in categories (Cs: cytoskeleton; Detox: oxidative metabolism/detoxification; Extmat: extracellular matrix; Imm: immunity; Met: metabolism; Ne: nuclear export; Nr: nuclear regulation; Pe: protein export; Pm: protein modification; Prot: proteosome machinery; Ps: protein synthesis machinery; S: secreted protein; St: signal transduction; Storage: storage protein; Te: transposable element; Tf: transcription factor; Tm: transcription machinery; Tr: transporters and channels) the JPCM5 predicted protein database was blasted using blastp. We automated annotation of proteins was based on a vocabulary of nearly 350 words found in matches to databases including Swissprot, Gene Ontology, KOG, Pfam, and SMART, Refseq‐invertebrates, and the diptera subset of the GenBank sequences obtained by querying diptera (organism) and retrieving all protein sequences.

### Data and statistical analyses

Principal component analyses (PCA) were performed with either the log_2_ TPMs or log_2_ fold change (LFC) data using the PAST3 software [27]. This software was also used to construct the bubble plots. Statistical analyses were carried out with PAST3 (multiple Mann Whitney U test) and Prism 7 (GraphPad Software Inc; all the other tests). Venn diagram results were obtained with Venny 2.1 (http://bioinfogp.cnb.csic.es/tools/venny/), and heat-maps/cluster analyses were obtained using the ClustVis tool [28] (https://biit.cs.ut.ee/clustvis/).

## Results

### Parasite growth and differentiation inside the sand fly midgut

**Fig 1:**
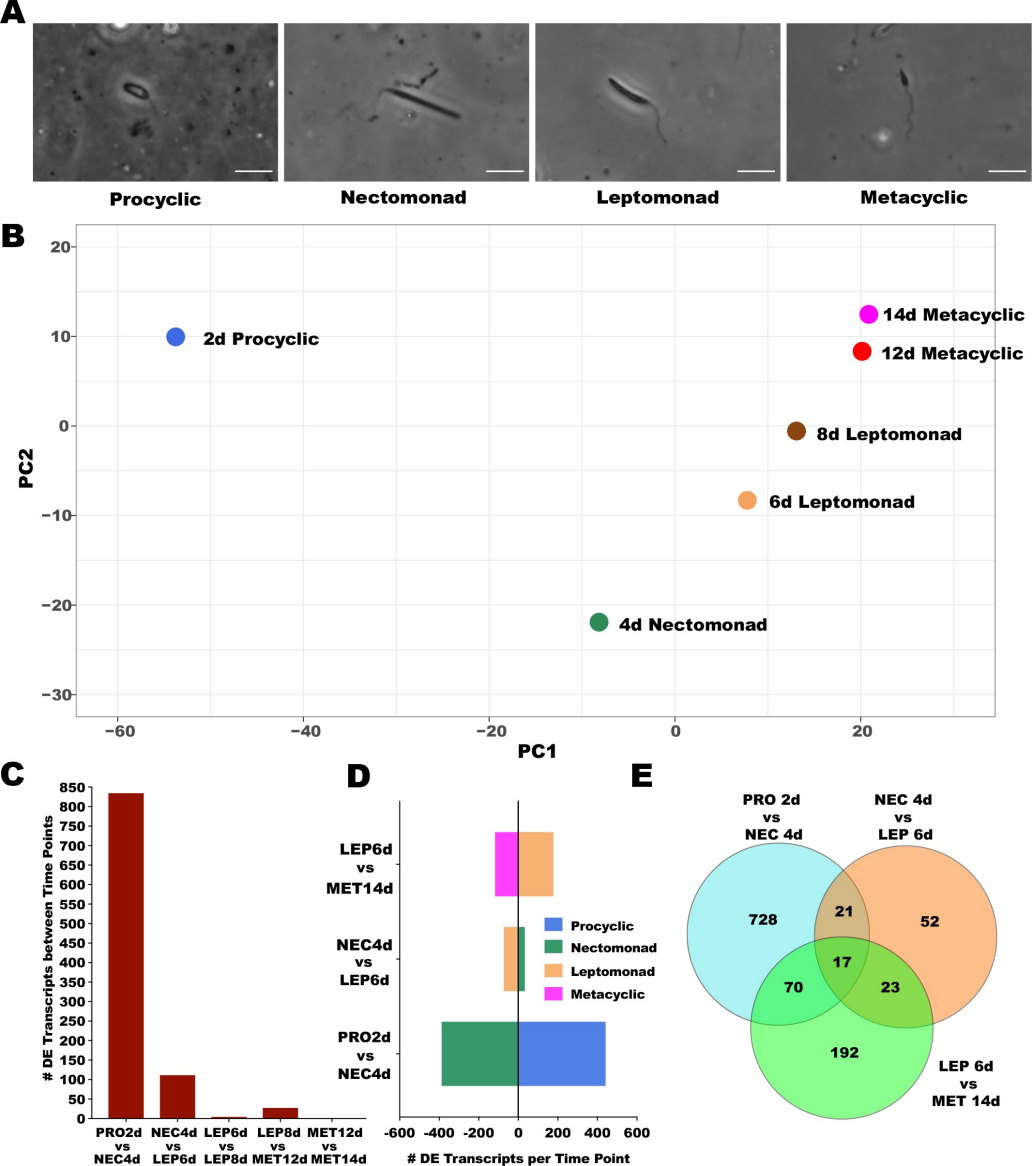
Parasite growth and overall analysis of *Leishmania* sequencing. **A.** Phase contrast images of the *Leishmania* parasites at different stages obtained from midguts at different time points. **B**. Principal component analysis (PCA) describing the position of each *Leishmania* time point in the expression space. Expression space was generated based on the log_2_ TPMs (transcripts per million) of the significantly differentially expressed transcripts across six time points. The Eigenvalues and % variance for PC1 and PC2 were 806.1 and 70.58% and 174.6 and 15.3%, respectively. **C**. Total number of differentially expressed transcripts between *Leishmania* time points. **D**. Enrichment of DE transcripts for each *Leishmania* stage in pairwise comparisons, as color coded in the legend. **E**. Venn diagrams depicting the number of DE transcripts unique and shared amongst pairwise comparisons of *Leishmania* stages. DE was considered significant for transcripts displaying FDR (false discovery rate) q-value lower than 0.05 and LFC (log_2_ fold change) either lower than -0.5 or higher than 0.5. PRO2d: procyclics at day 2. NEC4d: nectomonds at day 4. LEP6d: leptomonads 6 at days. LEP8d: leptomonads at 8 days. MET12d: metacyclics at day 12. MET14d: metacyclics at day 14.

The *Leishmania* parasite undergoes markedly different developmental stages inside the sand fly midgut including procyclics, nectomonads, leptomonads, and the infective stage, the metacyclics. We hypothesized that these different developmental stages display different patterns of transcriptional expression and that this information may define markers to molecularly distinguish distinct parasite stages. To test this hypothesis, we followed the growth and development of *L*. *infantum* parasites over time inside the midgut of the sand fly *Lu*. *longipalpis*. We dissected the sand fly midguts at six time points after infection where one of the different *Leishmania* stages is enriched (d2, d4, d6, d8, d12, and d14; Fig 1A; S1A Fig). The parasite growth in the sand fly midgut followed the expected pattern whereby at day 2 (2d), with blood still in the midgut, procyclic promastigotes were the prevailing parasite stage; at day 4 (4d), after blood digestion, there was a low level of parasites (median, 3,000 parasites) and consisted predominantly of nectomonad promastigotes; at day 6 and 8, parasites have multiplied (median, 16,000 parasites on day 6 and 35,000 on day 8) and mostly leptomonad promastigotes were observed (93% on day 6 and 70% leptomonad on day 8). On days 12 and 14, the predominant parasite stage were metacyclic promastigotes (73% on day 12 and 87% on day 14). By day 14, the parasites reached a median of 126,000 parasites per midgut and consisted predominantly of metacyclics (S1A and S1B Fig).

### Gene expression of the different *Leishmania* stages residing in the sand fly midgut

We performed RNAseq on RNA extracted from whole *Leishmania*-infected sand fly midguts opting not to purify *Leishmania* parasites to minimize transcriptional noise due to parasite manipulation, but focusing on time points where a specific *Leishmania* stage is predominant to detect differential expression among these stages. Experiments were carried out in three biological replicates for d4 (predominantly nectomonad promastigotes), d6 (predominantly leptomonad promastigotes), d8 (predominantly leptomonad promastigotes), d12 (predominantly metacyclic promastigotes), and d14 (predominantly metacyclic promastigotes), and two biological replicates for d2 (predominantly procyclic promastigotes). All RNAseq libraries gave rise to high quality data and robust expression levels and were used for further analyses. We mapped the trimmed reads to 8,150 protein-coding genes accounting for all protein-encoding genes identified in the *Leishmania* genome (JPCM5; https://www.sanger.ac.uk/resources/downloads/protozoa/leishmania-infantum.html). Of those, only one third had a known function (S2A Fig) represented by categories related to metabolism (met; 21%), signal transduction (st; 18%), protein synthesis machinery (ps; 14%), and protein modification (pm; 11%) (S2B Fig).

In order to assess the overall similarities in transcriptional profiles amongst *Leishmania* stages, we performed a Principal Component Analysis (PCA) with the overall transcriptional profiles of *Leishmania* obtained from all the libraries representing the different *Leishmania* stages (procyclics, nectomonads, leptomonads and metacyclics). This analysis is represented in a two-dimensional plot (Fig 1B; S3 Fig) and summarized in S1 Dataset. Interestingly, this unbiased analysis separated the different *Leishmania* stages to the different quadrants of the plot (Fig 1B). As the distance between points correlates with gene expression differences, the procyclic stage (parasites on day 2) were the most divergent population (Fig 1B, left top quadrant). The nectomonad stage (parasite samples from day 4), displayed the second most divergent expression pattern (Fig 1B, left bottom quadrant), followed by leptomonad-stage parasites (parasites present at day 6 and 8 samples) (Fig 1B, right bottom quadrant). The metacylic promastigotes (parasites on days 12 and 14) mapped closely together (Fig 1B, right top quadrant), indicating very similar gene expression profiles.

The overall pattern of gene expression observed in the PCA of the whole transcriptome (Fig 1B; q-value <0.05; -0.5 < LFC > 0.5) was further analyzed by comparing the differentially expressed (DE) genes between sequential *Leishmania* stages (Fig 1C; S2 Dataset). There were 836 differentially expressed genes between the procyclic stages (2d) and nectomonad stages (4d), indicating these two are highly distinct parasite stages dinamically transitioning from the small procyclic stage to a larger nectomonad stage (Fig 1C; S2 Dataset). The top 3 DE genes (in fold change) between these two stages were a putative calpain-like cysteine peptidase (LINJ_20_1220), a microtubule associated protein-like protein (LINJ_09_0190) and a d-isomer specific 2-hydroxyacid dehydrogenase-protein (LINJ_34_1510). There were 113 DE genes between nectomonad (4d) and leptomonad stages (6d) suggesting a less drastic transition between the two stages (Fig 1C; S2 Dataset). The top 3 DE genes between these two stages were a GP63 (LINJ_10_0530), a putative d-xylulose reductase (LINJ_33_0530) and glucose transporter (LINJ_36_6540).

As expected, only six genes displayed significant expression differences between leptomonads at day 6 (6d) and leptomonds at day 8 (8d) (Fig 1C) in accordance with their predominance at both timepoints. Between the multiplicative stage, the leptomonad (6d) and the non-multiplicative stage, the metacyclics (14d), there were 302 DE genes (Fig 1C; S2 Dataset), reflecting the major physical changes that occur as the parasite transition between these two stages (Fig 1A). The top 3 DE genes between the leptomonad and the metacyclic stages were a putative surface antigen protein 2 (LINJ.12.0665), an hypothetical protein (LINJ_04_0160) and another putative surface antigen protein 2 (LINJ.12.0666).

There were no differentially expressed genes when comparing metacyclics at day 12 (12d) and metacyclics at day 14 (14d; Fig 1C) suggesting that this parasite stage represents a very homogenous population at these timepoints. Even though a low number of parasites from preceding stages is likely present across most of the studied time points, the predominance of the procyclic stage on 2d, the nectomonad stage on 4d, the leptomonad stage on 6d and 8d, and the metacyclic stage on 12d and 14d (S1B Fig) was clearly reflected by gene expression differences between the different *Leishmania* stages (Fig 1C).

We performed a pairwise comparison of DE genes between *Leishmania* stages, and for the most part this analysis revealed an even number of up-regulated genes in each stage (Fig 1D; S2 Dataset). When comparing procyclic (2d) and nectomonad (4d) stages, 445 genes were up-regulated in the procyclic stage and 391 genes were up-regulated in the nectomonad stage (Fig 1D, green and blue bars). Between nectomonad (4d) versus leptomonad (6d) stages, 36 genes were up-regulated in the nectomonad stage and 77 genes were up-regulated in the leptomonad stage (Fig 1D, light brown and green bars). Between leptomonad and metacyclic stages, there were only 181 genes up-regulated in the leptomonad stage (6d) and only 121 genes up-regulated in the metacyclic stage (14d; Fig 1D, light brown and pink bars).

We further compared genes that were DE across multiple stages to those that were DE between only two stages (Fig 1E; S3 Dataset). For the most part, DE genes between two stages were more abundant than DE genes shared by multiple stages, highlighting the existence of transcriptional boundaries for each *Leishmani*a stage (Fig 1E; S4 Dataset).

**Fig 2:**
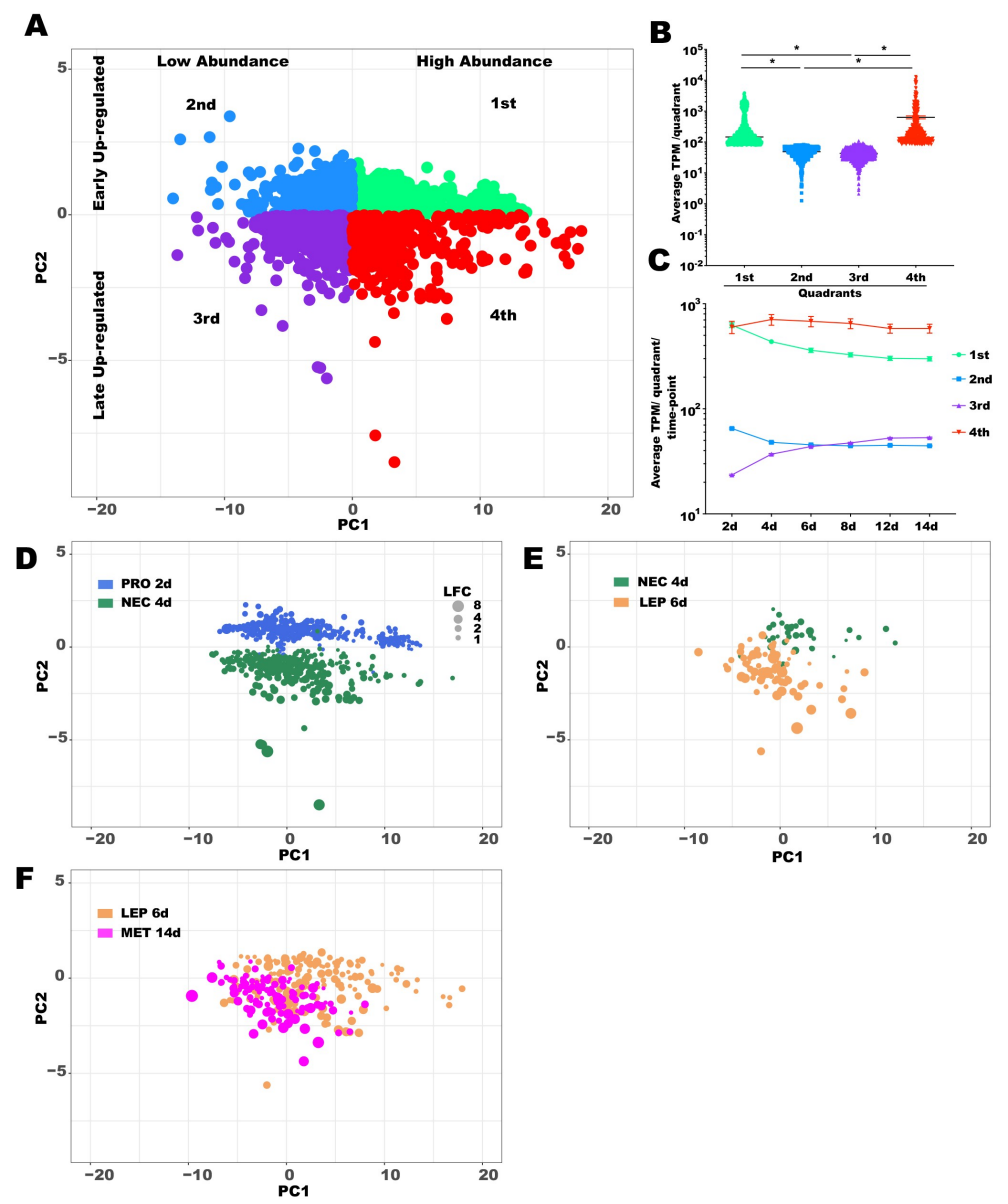
Analysis of differentially expressed (DE) transcript enrichment in different *Leishmania* stages. **A**. Principal Component Analysis (PCA) analysis of all the DE transcripts in all time points based on the log_2_ fold change (LFC) of every pairwise combination of *Leishmania* time points. Each quadrant in the expression space was label from 1^st^ to 4^th^ and the transcripts mapped to the respective quadrants were color coded in Spring Green (1^st^), Dodge Blue (2^nd^), Blue Violet (3^rd^), and Red (4^th^). The Eigenvalues and % variance for PC1 and PC2% were 20.69 and 95.35% and 0.68 and 3.15%, respectively. **B**. Expression analysis per quadrant. The average TPM across time points for every DE transcript mapped in each quadrant was plotted. Horizontal bars indicate median values and differences were statistically significant (* Mann Whitney test, p < 0.0001). Color coding as in A. **C**. Expression analysis per quadrant per time point. The average TPM for each time point for every DE transcript mapped in each quadrant was plotted. Mean TPM as shapes and SEM (standard error of mean) bars are depicted. Based on the differences observed in B and C, the quadrants in A were labeled to describe the up-regulated transcripts expressed in high and low abundance (as defined by PC1) and expressed early and late time points (as defined by PC2). **D-F**. *Leishmania* DE transcripts up-regulated in each stage mapped onto the expression space. D. Bubble plot mapping the procyclic up-regulated transcripts (Royal blue) and the nectomonad up-regulated ones (Sea green) on the transcriptional space. Scale in gray represents the log_2_ fold change corresponding to the diamenter of the bubbles. E. Bubble plot mapping the nectomonad up-regulated ones (Sea green) and the leptomonad up-regulated ones (Saddle brown) on the transcriptional space. F. Bubble plot mapping the leptomonad up-regulated ones (Saddle brown) and the metacyclic up-regulated ones (Fuchsia) on the transcriptional space. Differences were statistically significant at p < 0.001 (Chi-square test). DE was considered significant for transcripts displaying FDR q-value lower than 0.05 and LFC either lower than -0.5 or higher than 0.5. PRO2d: procyclics at day 2. NEC4d: nectomonds at day 4. LEP6d: leptomonads at 6 days. MET14d: metacyclics at day 14.

We then evaluated whether or not the *Leishmania* genes display different expression patterns throughout development by performing a PCA with all the differentially expressed genes (3,277 differentially expressed genes; q-value <0.05; -0.5 < LFC > 0.5; S4 Dataset; S4 Fig). Such DE genes account for all pairwise comparisons between the different *Leishmania* stages mapped onto a two dimensional space (Fig 2A). Furthermore, we described what the variability in components 1 and 2 account for, by averaging the transcriptional levels of the genes per quadrant and across time points. In fact, DE genes that mapped onto the first (Fig 2A, top right; Fig 2B, average TPM: 392) and fourth quadrants (Fig 2A, bottom right; Fig 2B, average TPM: 633.5) presented about a ten-fold higher average expression than those DE genes that mapped onto the second (Fig 2A, top left; Fig 2B, average TPM: 48.7) and third quadrants (Fig 2A, bottom left; Fig 2B, average TPM: 40.2; S4 Dataset); therefore, we define the PC1 (top quadrants) as a measure of transcriptional abundance. Further, the differentially expressed genes in the first and second quadrants were up-regulated in early time points, whereas those mapped onto the third and fourth quadrants were up-regulated in the late time points (Fig 2A and 2C; S4 Dataset), suggesting that PC2 accounted for temporal variability in transcription across stages (bottom quadrants).

Mapping the DE genes enriched in each *Leishmania* stage onto the expression space further underscored the unique expression profiles of the different *Leishmania* stages (Fig 2D–2F; S5A–S5C Fig; S2 Dataset). When comparing DE genes between the procyclic and nectomonad stages, the mapping showed that the procyclic-up-regulated genes were mostly early up-regulated whereas the nectomonad up-regulated genes were predominantly late up-regulated (Chi-square test, p < 0.001; Fig 2D; S5A Fig). When comparing the DE genes in nectomonad and leptomonad stages, we observed the nectomonad up-regulated genes encompassed genes that were expressed in high abundance and were early up-regulated (Fig 2E; S5B Fig). On the other hand, most of the leptomonad up-regulated genes belonged to the low abundance/late up-regulated genes (p < 0.001; Fig 2E; S5B Fig). For the DE genes up-regulated in either leptomonad or metacyclic stages (Fig 2F; S5C Fig), the maping pattern indicated that the metacyclic up-regulated genes belonged predominantly to the late up-regulated group of genes whereas the leptomonad-up-regulated genes displayed a more broad-spectrum expression pattern (p < 0.001; Fig 2F; S5C Fig).

**Fig 3:**
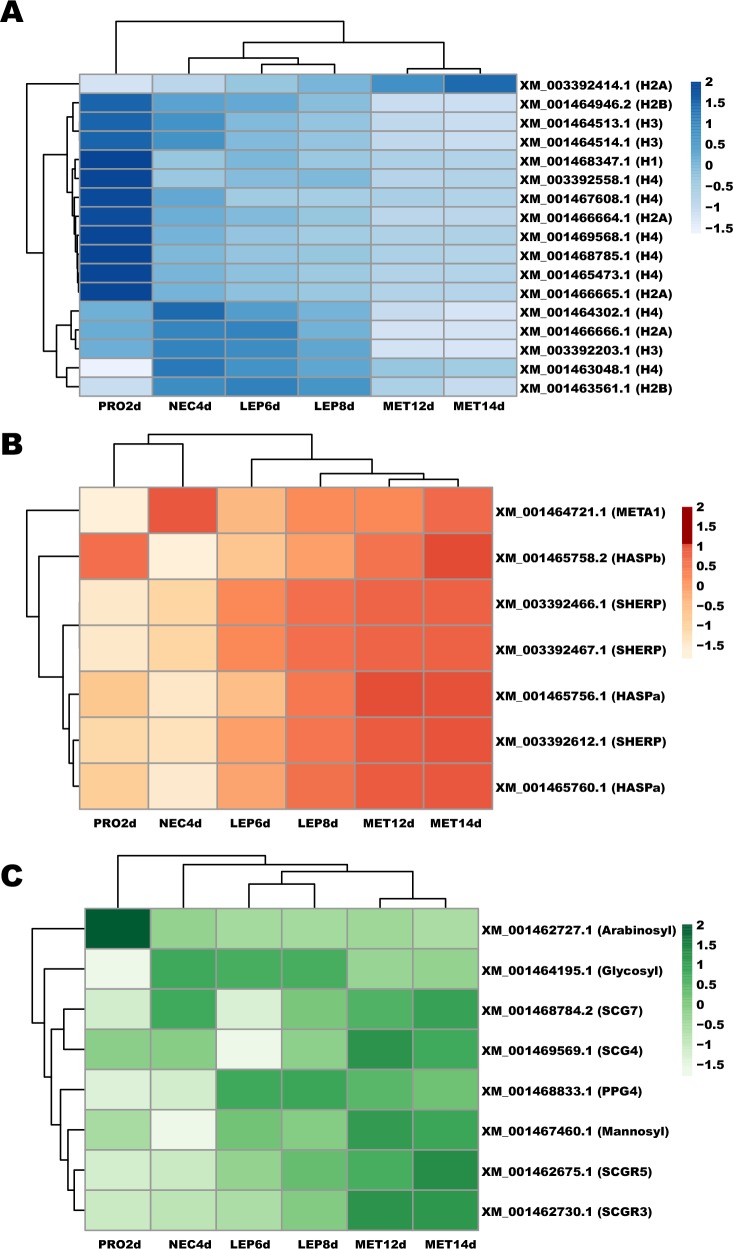
Heatmap depicting temporal expression of selected DE genes. **A.** Genes encoding histone proteins. H1: histone H1. H2A: histone H2A; H2B: histone H2B; H3: histone H3. H4: histone H4. **B.** Metacyclogenesis-related genes. HASPa: hydrophilic acylated surface protein a; HASPb: hydrophilic acylated surface protein b; SHERP: small hydrophilic endoplasmic reticulum-associated protein; META1: META domain-containing protein. **C.** Genes involved in phosphoconjugate sythesis. Arabinosyl: phosphoglycan beta 1,2 arabinosyltransferase; Glycosyl: glycosyltransferase family-like protein; Galactosyl: phosphoglycan beta 1,3 galactosyltransferase; Mannosyl: mannosyltransferase-like protein; PPG4: proteophosphoglycan; LPG3: glucose regulated protein 94. GenBank gene Ids and color intensity scale are also depicted on the left. PRO2d: procyclics at day 2; NEC4d: nectomonads at day 4; LEP6d: leptomonads at day 6; LEP8d: leptomonads at day 8; MET12d: metacyclics at day 12; MET14d: metacyclics at day 14.

Between-stage differences were also noticed for specific gene families displaying important roles during parasite growth and differentiation in the sand fly vector (Fig 3; S5 Dataset). For instance, the number of enriched histone genes, and their expression levels, gradually decreased from the procyclic to the metacyclic stage (Fig 3A; S5 Dataset). Genes encoding the small hydrophilic endoplasmic reticulum-associated protein (SHERP) and hydrophilic acylated surface protein a (HASPa), associated to metacylogenesis [29], were up-regulated in leptomonads compared to nectomonads (Fig 3B; S5 Dataset), and overall exhibited the highest expression in metacylics (Fig 3B). On the other hand, the gene encoding the META1 protein, also associated to metacylogenesis [29], was up-regulated as early as the nectomonad stage compared to procyclic (Fig 3B; S5 Dataset). Regarding the genes involved in the elongation of the glycoconjugate LPG, transcripts for a mannosyltrasferase and the galactosyltransferases (SCG4, SCG7, SCGR3, and SCGR5) were up-regulated in leptomonads and metacyclics, respectively (Fig 3C; S5 Dataset). A glycosyltransferase gene (LINJ_14_0500), involved in the addition of the LPG’s glucose side chains, was down-regulated in metacyclics (Fig 3C; S5 Dataset). Transcription of the gene *ppg4*, responsible for the synthesis of the glycoconjugate proteophosphoglycan, was up-regulated in leptomonads and metacyclics as compared to nectomonads or procyclics (Fig 3C; S5 Dataset).

**Fig 4:**
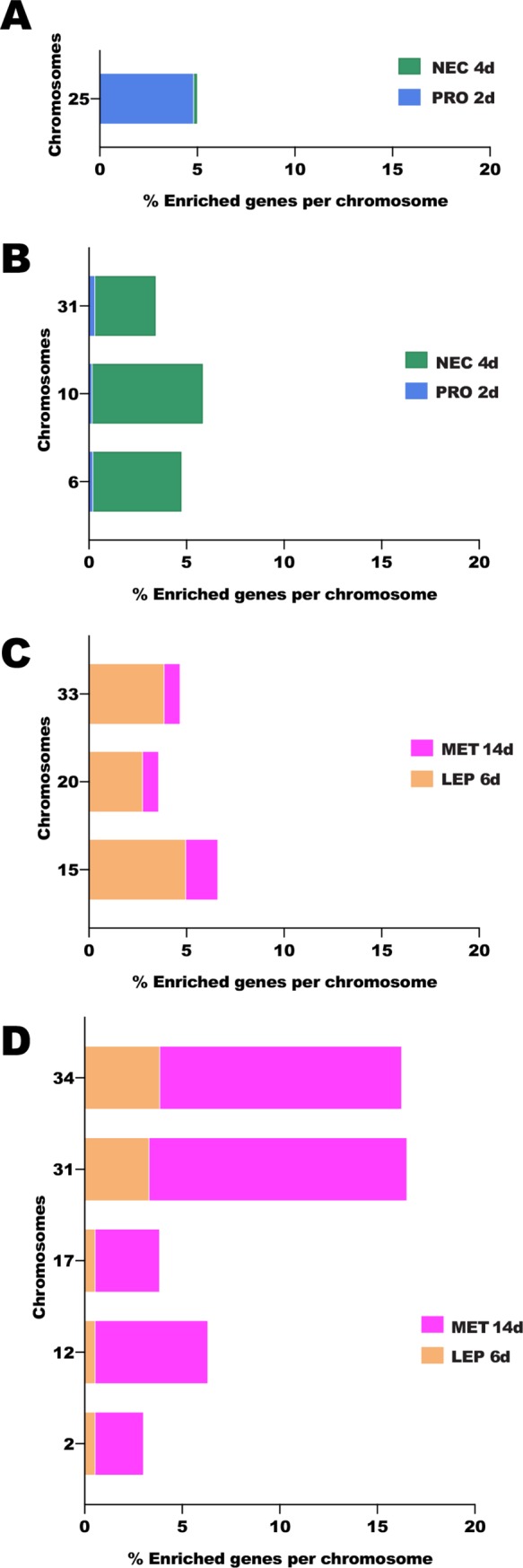
Chromosome displaying at least three-fold enrichment of DE genes across time *Leishmania* stages. **A.** Chromosomes displaying enrichment of DE genes from the procyclic to the nectomonad stage. **B.** Chromosomes exhibiting decrease in the proportion of DE genes from the procyclic to the nectomonad stage. **C.** Chromosomes displaying enrichment of DE genes from leptomonad to metacyclic stage. **D.** Chromosomes exhibiting decrease in the proportion of DE genes from the leptomonad to the metacyclic stage. PRO2d: procyclics at day 2. NEC4d: nectomonds at day 4. LEP6d: leptomonads at 6 days. MET14d: metacyclics at day 14.

Interestingly, the different stages of *Leishmania* parasites exhibited specific transcriptional enrichment across chromosomes (Fig 4; S6 Dataset). Differences in the proportion of enriched genes across chromosomes were statistically significant between procyclics and nectomonads as well as nectomonads and metacyclics (chi-square p < 0.0001) but not between nectomonads and leptomonads. Regarding the most strinking differences, there was a three-fold (or higher) enrichment of genes up-regulated in procyclics compared to nectomonads on chromosome 25 (Fig 4A). In contrast, a three-fold (or higher) enrichment of up-regulated genes in nectomonads was noticed on chromosomes 6, 10, and 31 for nectomonads compared to procyclics (Fig 4B). Between leptomonad and metacyclic stages, a three-fold (or higher) enrichment of up-regulated genes in leptomonads was seen on chromosomes 15, 20, and 33 for leptomonads compared to metacyclics (Fig 4C). On the other hand, five chromosomes (2, 12, 17, 31, and 34) displayed at least three-fold higher abundance of up-regulated genes in metacyclic compared to leptomonads (Fig 4D).

### Candidates for stage-specific molecular markers for *Leishmania infantum*

**Fig 5:**
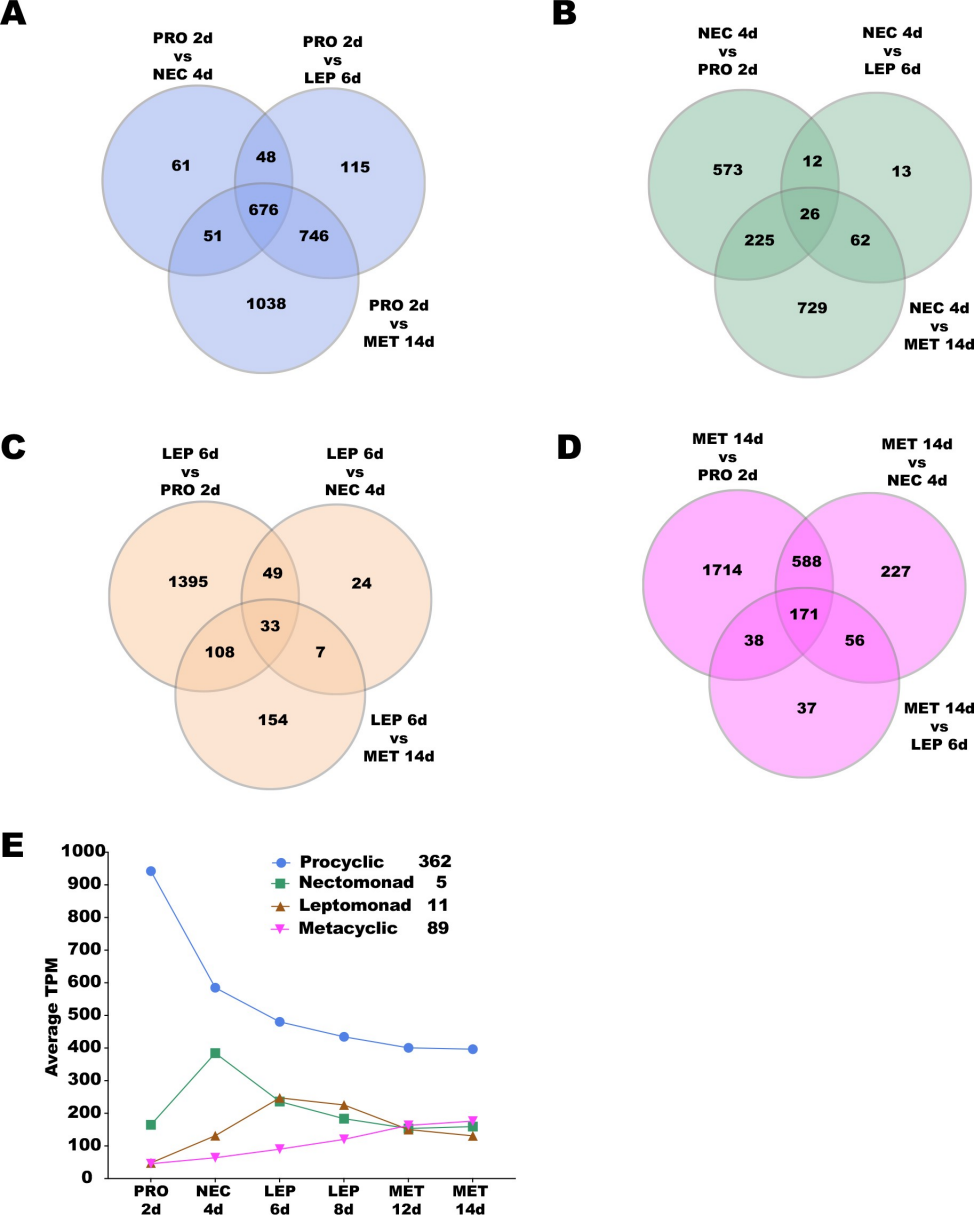
Candidate *Leishmania* stage-specific markers. Venn diagrams highlighting (in white) the numbers of DE genes between (**A**) procyclics, (**B**) nectomonad, (**C**) leptomonad, and (**D**) metacyclic. **E.** Overall expression profile patterns of the candidate *Leishmania* stage-specific markers. Number of candidate genes per stage are shown in the inset. PRO2d: procyclics at day 2. NEC4d: nectomonds at day 4. LEP6d: leptomonads 6 at days. LEP8d: leptomonads at 8 days. MET12d: metacyclics at day 12. MET14d: metacyclics at day 14.

The differential gene expression between *Leishmania* stages allowed for the identification of stage-specific molecular markers: genes predominantly expressed in one stage compared to all other stages (Fig 5). We initially searched for the genes that were DE between one stage and any other stage (Fig 5A–5D). Among these, we identified subsets of genes exhibiting stage-specific transcriptional enrichment, i.e. expression levels at LFC > 0.5 and q-value < 0.05 compared to any other stage (Fig 5E; S7 Dataset). Among those, 362 genes were up-regulated in procyclics, 5 genes presented a higher expression in nectomonads, 11 genes were up-regulated in leptomonads, and 89 genes displayed metacyclic-specific up-regulation (Fig 5E; S8 Dataset). Among stage-specific candidates, the genes encoding surface proteins are listed in Table 1, with the exception of the nectomonad stage, which was devoid of candidates. Among other markers, stage-specific genes included the ATPase alpha subunit and the ATP-binding cassette protein subfamily E in the procyclic stage; a hypothetical protein similar to a surface antigen-like protein and a putative ATG8/AUT7/APG8/PAZ2 in leptomonads; and the surface antigen protein 2, putative amastin-like surface protein, and leishmanolysin in metacyclics. In Table 2, we list the most promising stage-specific makers, encompassing the genes displaying the greatest transcriptional fold change differences to the subsequent stage in development.

**Table 1:** Selected stage-specific up-regulated-genes encoding membrane proteins.

Stage	GeneID	Locustag	NR best match	Description	e-value	log2 FC PRO2d vs NEC4d	log2 FC PRO2d vs LEP6d	log2 FC PRO2d vs MET14d
**PRO2d**	XM_001462976.2	LINJ_05_0510	XP_001463014.1	ATPase alpha subunit	0	0.777	0.896	1.197
	XM_001465345.1	LINJ_21_0770	XP_001465382.1	ATP-binding cassette protein subfamily E, member 1	0	0.854	1.243	1.430
						**log2 FC PRO2d vs LEP6d**	**log2 FC NEC4d vs LEP6d**	**log2 FC LEP6d vs MET14d**
**LEP8d**	XM_001462819.1	LINJ_04_0180	XP_001462856.1	surface antigen-like protein	0	-2.862	-0.888	1.435
	XM_001462818.1	LINJ_04_0170	XP_001462855.1	surface antigen-like protein	0	-2.869	-0.763	1.308
	XM_001462821.1	LINJ_04_0200	XP_001462858.1	surface antigen-like protein	0	-1.915	-1.500	1.080
	XM_001463438.1	LINJ_09_0180	XP_001463475.1	putative ATG8/AUT7/APG8/PAZ2	1E-90	-2.343	-0.739	0.890
						**log2 FC PRO2d vs MET14d**	**log2 FC NEC4d vs MET14d**	**log2 FC LEP6d vs MET14d**
**MET14d**	XM_001468504.1	LINJ_34_1710	XP_001468541.1	putative amastin-like surface protein	1.00E-136	-1.188	-1.221	-0.639
	XM_001468506.2	LINJ_34_1720	XP_001468543.2	putative amastin-like surface protein	0	-1.090	-0.958	-0.705
	XM_001468449.1	LINJ_34_1150	XP_001468486.1	putative amastin-like surface protein	7.00E-139	-1.694	-0.842	-0.719
	XM_001468507.1	LINJ_34_1730	XP_001468544.1	putative amastin-like surface protein	6.00E-138	-0.957	-0.974	-0.798
	XM_001468501.1	LINJ_34_1680	XP_001468538.1	putative amastin-like surface protein	2.00E-149	-1.476	-1.135	-0.893
	XM_001468436.1	LINJ_34_1020	XP_001468473.1	putative amastin-like surface protein	7.00E-123	-1.696	-0.801	-0.659
	XM_001468503.1	LINJ_34_1700	XP_001468540.1	putative amastin-like surface protein	6.00E-136	-1.681	-1.651	-1.211
	XM_003392619.1	LINJ_28_0610	XP_003392668.1	putative leishmanolysin	0	-2.214	-1.447	-0.692
	XM_001463664.2	LINJ_10_0520	XP_001463701.2	GP63, leishmanolysin	0	-2.896	-1.470	-0.707
	XM_001463660.2	LINJ_10_0530	XP_001463697.2	GP63, leishmanolysin	0	-4.894	-3.188	-1.206
	XM_003392666.1	LINJ_31_1850	XP_003392714.1	amino acid permease	0	-1.586	-1.535	-0.870
	XM_003392664.1	LINJ_31_1810	XP_003392712.1	amino acid permease	0	-2.249	-2.161	-0.762
	XM_001469645.1	LINJ_36_3500	XP_001469682.1	hypothetical transmembrane protein	0	-1.220	-1.044	-0.531
	XM_001462682.1	LINJ_02_0270	XP_001462719.1	LOW QUALITY PROTEIN: putative ABC1 transporter	0	-1.569	-1.184	-1.004
	XM_003392265.1	LINJ.12.0662	XP_003392313.1	putative surface antigen protein 2	2.00E-155	-2.536	-1.736	-1.170
	XM_001463964.2	LINJ.12.0670	XP_001464001.2	putative surface antigen protein 2	0	-3.113	-2.369	-1.261
	XM_003392269.1	LINJ.12.0666	XP_003392317.1	putative surface antigen protein 2	0	-3.771	-2.919	-1.498
	XM_003392274.1	LINJ.12.0690	XP_003392322.1	surface antigen protein 2 precursor	0	-3.653	-1.558	-1.105

**Table 2:** Selected top stage-specific markers displaying the highest fold change compared to the next stage.

Stage	GeneID	Locustag	NR best match	Description	e-value	log2 FC PRO2d vs NEC4d	log2 FC PRO2d vs LEP6d	log2 FC PRO2d vs MET14d
**PRO2d**	XM_001463562.1	LINJ_09_0660	XP_001463599.1	conserved hypothetical protein	7.00E-143	2.050	1.635	0.983
	XM_001469684.1	LINJ_09_1420	XP_001469721.1	conserved hypothetical protein	0	1.939	1.315	1.694
	XM_001466644.1	XM_001466644.1	XP_001466681.1	conserved hypothetical protein	0	1.772	1.778	2.234
	XM_001470194.1	LINJ_28_2060	XP_001470231.1	putative zinc transporter	0	1.565	2.237	2.230
	XM_003392629.1	LINJ_28_2060	XP_001470231.1	putative zinc transporter	0	1.565	2.237	2.230
	XM_001463906.2	LINJ_29_1600	XP_001463943.2	conserved hypothetical protein	0	1.499	1.043	1.678
	XM_001468347.1	LINJ_33_3390	XP_001468384.1	h1 histone-like protein	4.00E-83	1.470	1.229	2.101
	XM_001467545.1	LINJ_31_2650	XP_001467582.1	ubiquinol-cytochrome-c reductase-like protein	5.00E-44	1.456	1.769	2.260
	XM_003392567.1	LINJ_26_0990	XP_003392615.1	hypothetical protein LINJ_26_0990	2.00E-40	1.453	1.715	0.857
	XM_001466147.1	LINJ_25_1530	XP_001466184.1	cyclin	0	1.449	1.143	1.718
						**log2 FC PRO2d vs NEC4d**	**log2 FC NEC4d vs LEP6d**	**log2 FC NEC4d vs MET14d**
**NEC4d**	XM_001462831.1	LINJ_04_0300	XP_001462868.1	putative beta-fructofuranosidase	0	-0.664	0.930	2.124
	XM_001466274.1	LINJ_27_0430	XP_001466311.1	putative ribokinase	0	-0.805	0.853	0.610
	XM_003392396.1	LINJ_20_1730	XP_003392444.1	putative N-acyl-L-amino acid amidohydrolase	0	-2.658	0.649	1.925
	XM_001464164.1	LINJ_14_0180	XP_001464201.1	metallo-peptidase, Clan MA(E), Family M32	0	-0.793	0.607	1.132
	XM_001462905.1	LINJ_04_1030	XP_001462942.1	conserved hypothetical protein	1.00E-136	-1.149	0.505	1.236
						**log2 FC PRO2d vs LEP6d**	**log2 FC NEC4d vs LEP8d**	**log2 FC LEP8d vs MET14d**
**LEP 8d**	XM_001462819.1	LINJ_04_0180	XP_001462856.1	surface antigen-like protein	0	-2.863	-0.889	1.435
	XM_001462818.1	LINJ_04_0170	XP_001462855.1	surface antigen-like protein	0	-2.870	-0.764	1.309
	XM_001466805.1	LINJ_30_0290	XP_001466842.1	hypothetical protein, unknown function	0	-0.963	-0.595	1.102
	XM_001462821.1	LINJ_04_0200	XP_001462858.1	surface antigen-like protein	0	-1.916	-1.500	1.081
	XM_003392407.1	LINJ_21_1100	XP_003392455.1	putative mis-match repair protein	0	-1.930	-0.657	0.900
	XM_001463438.1	LINJ_09_0180	XP_001463475.1	putative ATG8/AUT7/APG8/PAZ2	1.00E-90	-2.343	-0.739	0.891
	XM_001470568.1	LINJ_26_2710	XP_001470605.1	hypothetical protein, unknown function	0	-2.445	-0.723	0.885
	XM_001464501.1	LINJ_16_0500	XP_001464538.1	conserved hypothetical protein	3.00E-142	-1.433	-0.605	0.867
	XM_001465134.2	LINJ_20_1220	XP_001465171.2	putative calpain-like cysteine peptidase	0	-9.404	-0.903	0.781
	XM_001468097.1	LINJ_33_0530	XP_001468134.1	putative d-xylulose reductase	0	-0.833	-1.751	0.701
						**log2 FC PRO2d vs MET14d**	**log2 FC NEC4d vs MET14d**	**log2 FC LEP8d vs MET14d**
**MET14d**	XM_003392269.1	LINJ.12.0666	XP_003392317.1	putative surface antigen protein 2	0	-3.771	-2.920	-1.498
	XM_001466148.1	LINJ_25_1540	XP_001466185.1	calpain family cysteine protease-like protein	0	-3.209	-2.614	-1.303
	XM_001463964.2	LINJ.12.0670	XP_001464001.2	putative surface antigen protein 2	0	-3.114	-2.369	-1.262
	XM_001467534.1	LINJ_31_2540	XP_001467571.1	putative lipase	0	-2.277	-2.208	-1.221
	XM_001468503.1	LINJ_34_1700	XP_001468540.1	putative amastin-like surface protein	6.00E-136	-1.681	-1.652	-1.212
	XM_001463660.2	LINJ_10_0530	XP_001463697.2	GP63, leishmanolysin	0	-4.895	-3.188	-1.207
	XM_001464983.1	LINJ_19_0570	XP_001465020.1	conserved hypothetical protein	0	-3.217	-2.002	-1.175
	XM_003392265.1	LINJ.12.0662	XP_003392313.1	putative surface antigen protein 2	2.00E-155	-2.537	-1.736	-1.170
	XM_003392196.1	LINJ_08_1220	XP_003392244.1	hypothetical protein, unknown function	0	-0.604	-1.078	-1.130
	XM_003392274.1	LINJ.12.0690	XP_003392322.1	surface antigen protein 2 precursor	0	-3.654	-1.559	-1.106

## Discussion

In a recent study, the transcriptome of the *Leishmania major* midgut stages were compared with the mammalian amastigote stage, sheding light on the multiple biological processes leading to parasite differentiation [18]. The transcriptome of the midgut harvested *Leishmania* were also very similar to parasites harvested from culture [18]. In the present study, we hypothesized that different *Leishmania* developmental stages inside the sand fly midgut would have a different pattern of transcriptional expression, and that this information could help us to define molecular markers for each of these parasite stages. Our results of high-throughput RNA sequencing of the *L*. *infantum* stages in the midgut of the sand fly *L*. *longipalpis* clearly defined the transcriptional boundaries between the different *Leishmania* stages as well as identified gene candidates for *Leishmania* stage-specific molecular markers. It worthy to point out that by using this strategy, we have overlooked a small population of parasites attached to the stomodeal valve, the haptomonad parasites. Future studies will be needed to address the expression profile of this small yet biologically relevant stage. This will require more refined techniques, such as tissue dissociation and single-cell RNA-Seq. Further studies are also needed to account for the transcriptome profile of the newly described *Leishmania* stage, the retroleptomonads.

Initial microarrays studies have identified < 5% DE genes between *Leishmania* amastigotes and promastigotes from culture, contrasting to 15% differential expression at the proteomic level [13]. Such a disconnect between transcription and translation suggested that the *Leishmania* genome was constitutively transcribed, and that the control of gene expression was carried out post-transcriptionally at the level of RNA processing and/or translation [13]. In the current work, using statistical settings to detect differential gene expression (q-value < 0.05 and -0.5 < LFC > 0.5), we have identified 3,277 DE genes amongst all the time points analyzed, which represent 40.2% of the protein-coding genes in the *L*. *infantum* genome. These high levels of gene expression plasticity indicates that the *Leishmania* stages, exhibiting different morphologies in the sand fly midgut, undergo stage-specific changes at the RNA level. These numbers are comparable to previous work studying culture promastigotes versus macrophage amastigotes using less stringent DE statistics [15–17].

Amongst the DE genes between *L*. *infantum* midgut stages, we identified genes that were expressed at overall high or low abundance, but also we identified genes up-regulated at early compared to later stages. Also, such sets of DE genes were enriched at different proportions in the different stages. Together, these studies point to the existence of gene expression plasticity at the RNA level between *Leishmania* stages, suggesting that control of gene expression during *Leishmania* differentiation needs to be further explored [12].

The different stages of *L*. *infantum* promastigotes also displayed chromosome-specific patterns of enrichment or reduction of DE genes. *L*. *infantum* tetraploid chromosome 31 [1] displayed a gradual increase in the proportion of upregulated genes from procyclics to nectomonads and from leptomonads to metacyclics. This may be a mechanism to increase gene expression differences between stages as was reported for *L*. *mexicana* amastigotes, where the expression of genes located on the tetraploid chromosome 30, a homolog to chromosome 31 in *L*. *infantum*, was enriched [17]. In fact, other *L*. *infantum* chromosomes presented an increase or a decrease in the proportion of DE genes as *L*. *infantum* differentiated from one stage to the next. This phenomenon encompassed not only polysomic (chromosomes 6, 17, 25, 31, and 33) but also multiple disomic (chromosomes 2, 10, 12, 15, 20, and 34) chromosomes [1] across *L*. *infantum* stages, ruling out chromosomal somy level as a determinant of the differences in the proportions of DE genes detected across *Leishmania* stages. It is noteworthy that the genes differentially expressed during the *Leishmania* ontogeny, which must be hardwired, are also housed on the genetically more stable disomic chromosomes less prone to genetic divergence than their aneuploid counterparts [4].

RNAseq analysis of gene expression between *Leishmania* stages also detected a stronger correlation between gene and protein expression, which had previously been neglected by microarray analysis [13]. Multiple *L*. *infantum* histones have been shown to be down-regulated during metacyclogenesis *in vitro* [30, 31]. Similarly, multiple histone transcripts were consistently down-regulated throughout *L*. *infantum* differentiation in *L*. *longipalpis* midguts in this study, and in *L*. *major* developing in *P*. *papatasi* [18]. These findings are in line with the observation that histone gene expression decreases in differentiated cells of higher eukaryotes [32]. The major surface glycan–the lipophosphoglycan (LPG)–of *L*. *infantum* exhibits glucose side chains, which are maintained during metacyclogenesis [33]. The sugar transferase genes are responsible for the backbone elongation and side-chain decoration of LPG during *Leishmania* metacyclogenesis [34, 35]. Consistent with such a pattern, the glycosyltransferase gene is up-regulated in nectomonads, when LPG is present in high abundance on the parasite’s surface. Similarly, we have identified mRNA up-regulation of galactosyl- and mannosyltransferases in the leptomonad and metacyclic stages, consistent with elongation of LPG in the metacyclic stage [36]. A similar phenomenon was observed for genes linked to *Leishmania* differentiation into infective metacyclics, such as SHERP and HASPa [29, 37]. In accordance with the stationary-phase specific expression of such proteins, the correspondent transcripts are up-regulated in the leptomonad and metacyclic stages. Up-regulation of such genes was also observed in *L*. *major* metacyclics harvested from sand flies [18]. Along the same lines, one of the genes encoding the *Leishmania* proteophosphoglycan, PPG4 [38, 39], is up-regulated in the leptomonad and maintained at similar levels in the metacyclic stage. At these stages, *Leishmania* secretes a proteophosphoglycan-rich plug in the anterior midgut, which allows these parasites to be regurgitated onto the skin upon sand fly feeding [9, 40].

One of the gaps in *Leishmania* research is the lack of stage-specific molecular markers and their potential usefulness in understanding the biology of parasite transmission and infection as recently shown by Giraud et al [41]. By unveiling the transcriptional boundaries between *L*. *infantum* stages, this study provides a catalogue of candidates for stage-specific molecular markers that can be tested alone or in combination in in-situ hybridization and Real-Time PCR studies. Such markers will allow the identification of different parasite stages from laboratory culture and vectors, which is important in vector competence and epidemiological studies. Amongst the stage-specific markers, some of which encode surface proteins might facilite the development of monoclonal antibodies and purification of different stages for functional studies. Furthermore, finding that genes encoding surface proteins are enriched in different *Leishmania* stages further supports the fact that surface proteins were one of the principal innovations in the evolution of trypanosomatids [42].

## Supporting information

S1 Fig Parasite growth. **A.** Total number of parasites in different time points from single dissected midguts. Horizontal bars indicate median. Pool data from three independent infections. **B**. Proportion of the most predominant *Leishmania* stage obtained in each time point. PRO2d: procyclics at day 2. NEC4d: long nectomonds at day 4. LEP6d: leptomonads 6 at days. LEP8d: leptomonads at 8 days. MET12d: metacyclics at day 12. MET14d: metacyclics at day 14.
(TIF)

S2 Fig Molecular Functions of the *Leishmania* genes. **A**. Pie chart depicts the overall proportion of transcripts displaying known molecular functions (Known) and orphan sequences (Unknown). **B**. Pie chart displaying the proportion of genes belonging to different molecular functions. Cs: cytoskeleton; Detox: oxidative metabolism/detoxification; Extmat: extracellular matrix; Imm: immunity; Met: metabolism; Ne: nuclear export; Nr: nuclear regulation; Pe: protein export; Pm: protein modification; Prot: proteosome machinery; Ps: protein synthesis machinery; S: secreted protein; St: signal transduction; Storage: storage protein; Te: transposable element; Tf: transcription factor; Tm: transcription machinery; Tr: transporters and channels.
(TIF)

S3 Fig Principal component analysis (PCA) describing the position of each replicate of each *Leishmania* time point in the expression space. Expression space was generated based on the log_2_ TPMs (transcripts per million) using all expressed transcripts across six time points. The Eigenvalues and % variance for PC1 and PC2 were 601.2 and 43.02% and 166.9 and 11.94%, respectively. 2d: procyclics at day 2. 4d: long nectomonds at day 4. 6d: leptomonads 6 at days. 8d: leptomonads at 8 days. 12d: metacyclics at day 12. 14d: metacyclics at day 14. Numbers after time points, for instance 2d.2, indicate replicate number.
(TIF)

S4 Fig Heatmap depicting the expression profiles of the 3,277 DE genes across the six time points. (TIF)

S5 Fig Doughnut chart showing the proportion of enriched transcripts between different stages. (A-C) Procyclic (A), long nectomonad (B) and leptomonad (C) stages per quadrant (inner circles) and the proportion of DE transcripts per quadrant in long nectomonad (A), leptomonad (B), and metacyclic (C) stages (outer circles). Differences were statistically significant at p < 0.0001 (Chi-square test).
(TIF)

S1 Dataset Expression levels (log2 average TPMs per time point) of all genes and PCA coordinates for each time point and for each replicate of each time point. PCA output: Eigenvalue and % variance. TPM: transcripts per million.
(XLSX)

S2 Dataset Differential expressed genes between *Leishmania* stages. Gene ID number, Principal Component Analysis coordinates, log_2_ TMP values (transcripts per million), gene annotation information, and counts of TMPs and reads. **(PRO2d vs NEC4d)** Procyclic over nectomonad comparison. **(NEC4d vs LEP6d)** Nectomonad over leptomonad comparison. (**LEP6d vs MET14d)** Leptomonad over metacyclic comparison.
(XLSX)

S3 Dataset Unique and shared DE genes between stages, as in the Venn diagram (Fig 1D). Gene ID number, log_2_ Fold change, gene annotation information, and counts of TMPs (transcripts per million) and reads. Positive fold change values indicate enrichment in the former whereas negative fold change values point to enrichment in the later stage.
(XLSX)

S4 Dataset Overall differential expressed genes across *Leishmania* stages and DE genes per PCA quadrant. Gene ID number, Principal Component Analysis coordinates, log_2_ TMP values (transcripts per million), gene annotation information, and counts of TMPs and reads.
(XLSX)

S5 Dataset Expression profiles of specific *Leishmania* genes of known function. Histones, metacyclogenesis (META1, SHERP, HASPa, HASPb), sugar transferases (glycosyl-, mannosyl-, and galactosyltransferases), and proteophosphoglycan (PPG). Gene ID number, fold change (log2), q-value (padj), gene annotation information (NR best match), e-values, TMP values (log2), average TMPs among replicates. **(PRO2d vs NEC4d)** Procyclic over nectomonad comparison. **(NEC4d vs LEP6d)** Nectomonad over leptomonad comparison. **(LEP6d vs MET14d)** Leptomonad over metacyclic comparison. Regarding fold changes, positive values indicate enrichment in the former whereas negative values point to enrichment in the later stage.
(XLSX)

S6 Dataset DE genes of each chromosome enriched in each different *Leishmania* stages. (XLSX)

S7 Dataset DE genes between one *Leishmania* stage and any other stage. PRO2d: procyclics at day 2. NEC4d: nectomonds at day 4. LEP6d: leptomonads 6 at days. LEP8d: leptomonads at 8 days. MET12d: metacyclics at day 12. MET14d: metacyclics at day 14.
(XLSX)

S8 Dataset Candidate markers of each *Leishmania* stage. NEC4d: nectomonds at day 4. LEP6d: leptomonads 6 at days. LEP8d: leptomonads at 8 days. MET12d: metacyclics at day 12. MET14d: metacyclics at day 14. TPM: transcripts per million. SD: standard deviation. SEM: standard error of the mean.
(XLSX)
