# Supplementary figures and images for: Distinct gene expression patterns in vector-residing Leishmania infantum identify parasite stage-enriched markers
Source: PLoS Negl Trop Dis. 2020 Mar 3;14(3):e0008014. doi: 10.1371/journal.pntd.0008014 (PMC7053709; doi:10.1371/journal.pntd.0008014)

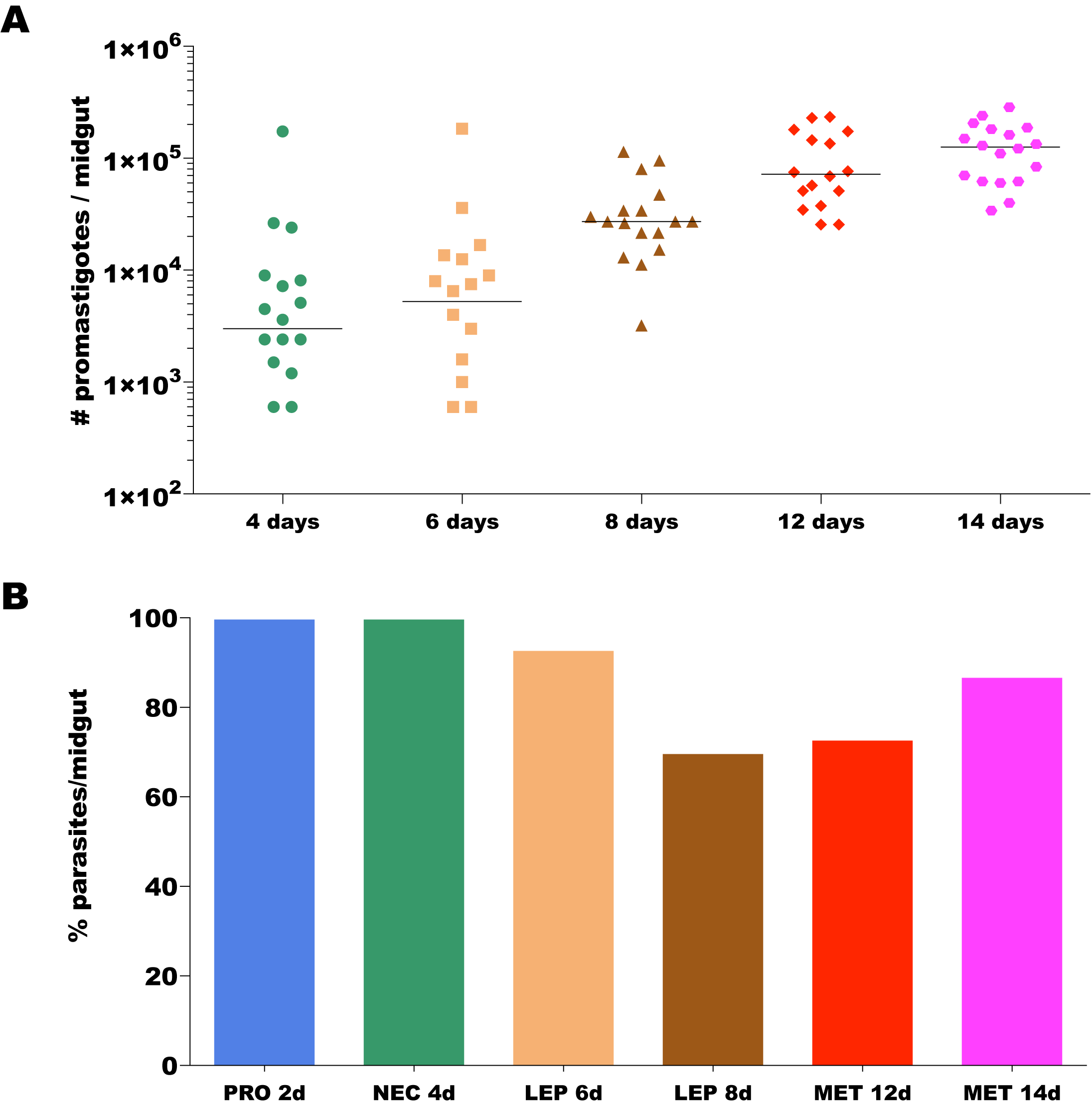

Supplement: S1 Fig — A. Total number of parasites in different time points from single dissected midguts. Horizontal bars indicate median. Pool data from three independent infections. B. Proportion of the most predominant Leishmania stage obtained in each time point. PRO2d: procyclics at day 2. NEC4d: long nectomonds at day 4. LEP6d: leptomonads 6 at days. LEP8d: leptomonads at 8 days. MET12d: metacyclics at day 12. MET14d: metacyclics at day 14. (TIF) [file pntd.0008014.s001.tif]

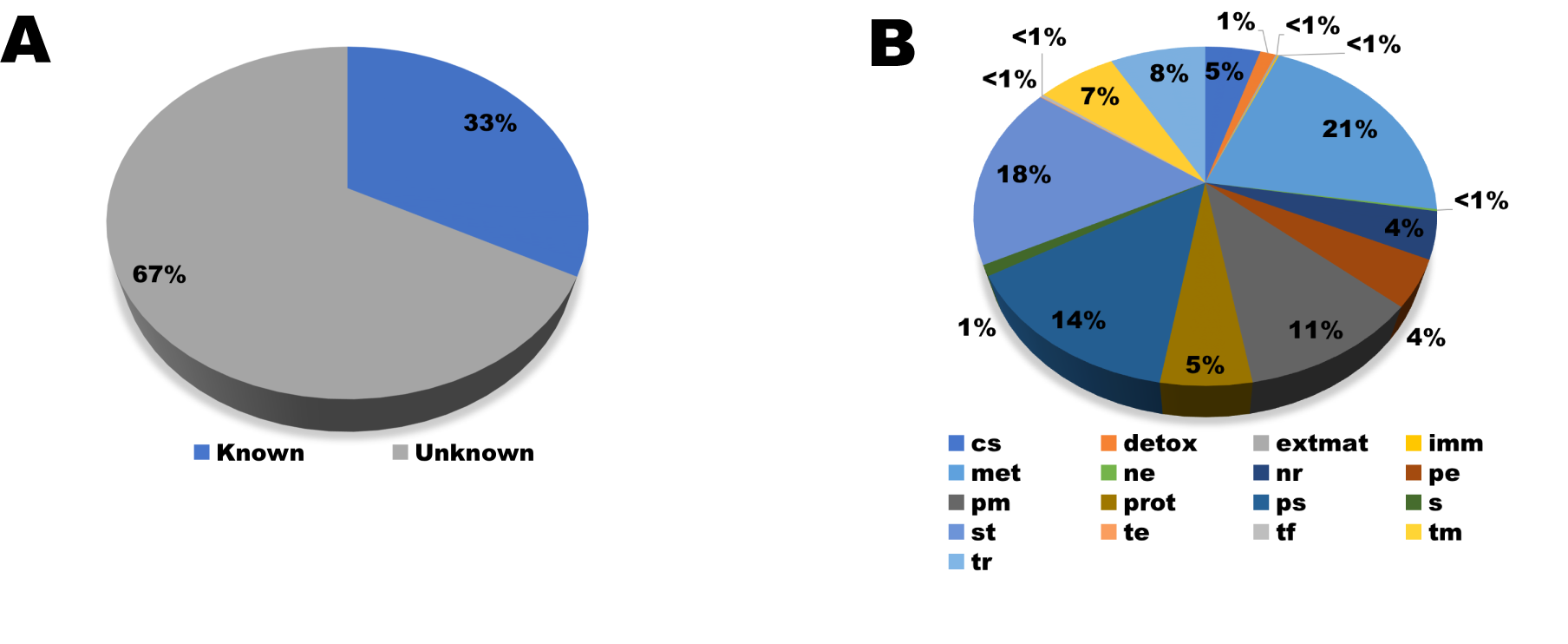

Supplement: S2 Fig — A. Pie chart depicts the overall proportion of transcripts displaying known molecular functions (Known) and orphan sequences (Unknown). B. Pie chart displaying the proportion of genes belonging to different molecular functions. Cs: cytoskeleton; Detox: oxidative metabolism/detoxification; Extmat: extracellular matrix; Imm: immunity; Met: metabolism; Ne: nuclear export; Nr: nuclear regulation; Pe: protein export; Pm: protein modification; Prot: proteosome machinery; Ps: protein synthesis machinery; S: secreted protein; St: signal transduction; Storage: storage protein; Te: transposable element; Tf: transcription factor; Tm: transcription machinery; Tr: transporters and channels. (TIF) [file pntd.0008014.s002.tif]

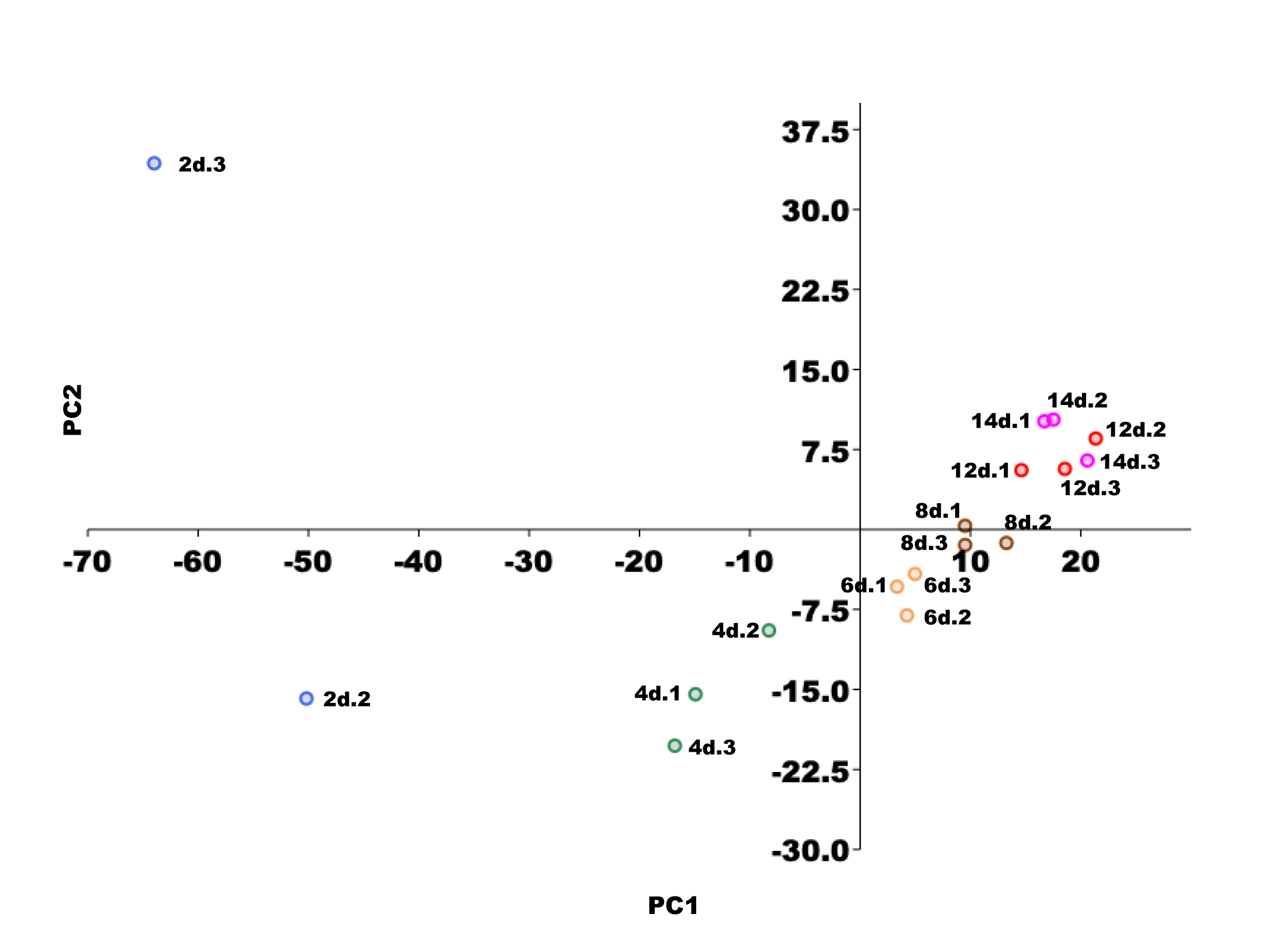

Supplement: S3 Fig — Expression space was generated based on the log2 TPMs (transcripts per million) using all expressed transcripts across six time points. The Eigenvalues and % variance for PC1 and PC2 were 601.2 and 43.02% and 166.9 and 11.94%, respectively. 2d: procyclics at day 2. 4d: long nectomonds at day 4. 6d: leptomonads 6 at days. 8d: leptomonads at 8 days. 12d: metacyclics at day 12. 14d: metacyclics at day 14. Numbers after time points, for instance 2d.2, indicate replicate number. (TIF) [file pntd.0008014.s003.tif]

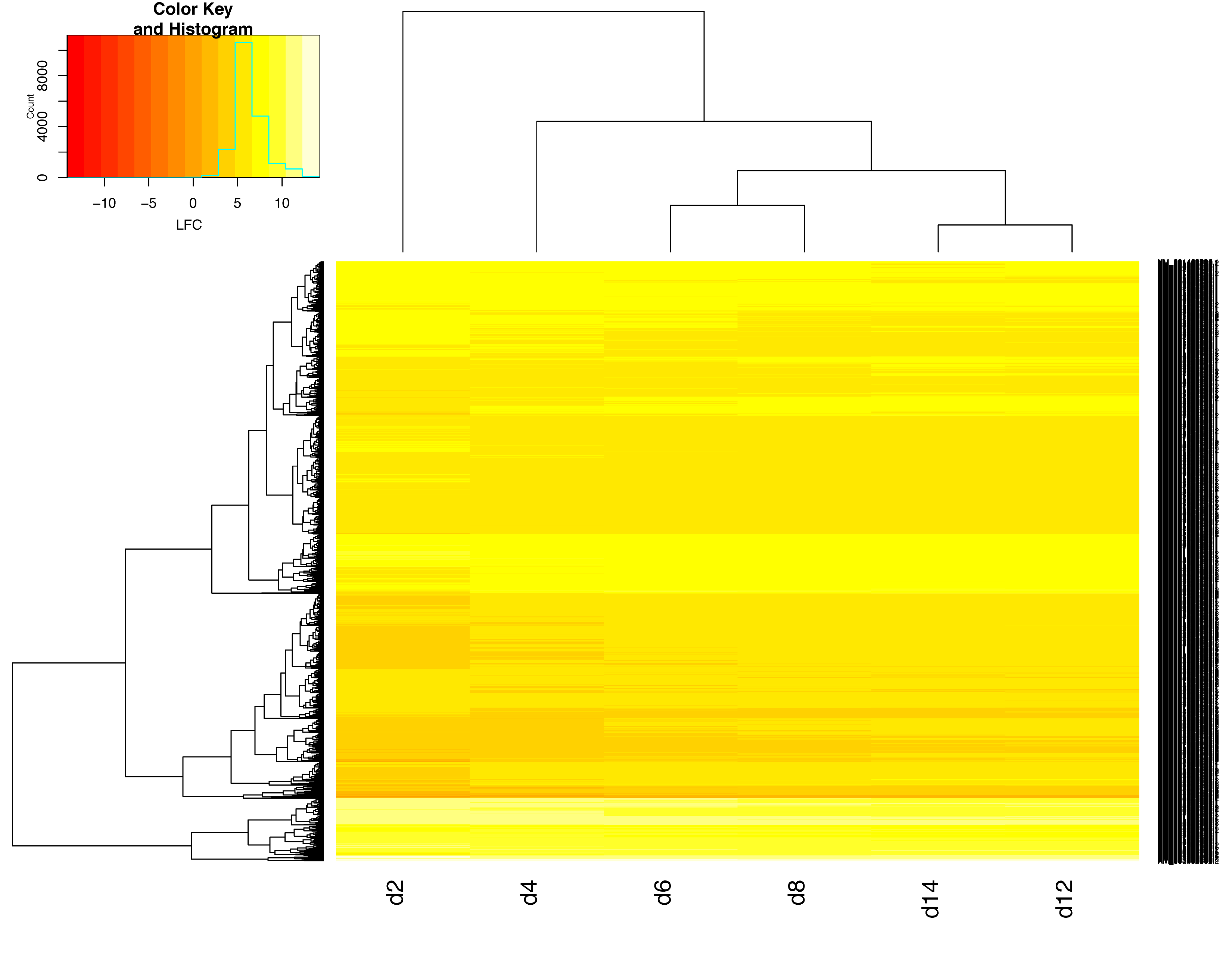

Supplement: S4 Fig — (TIF) [file pntd.0008014.s004.tif]

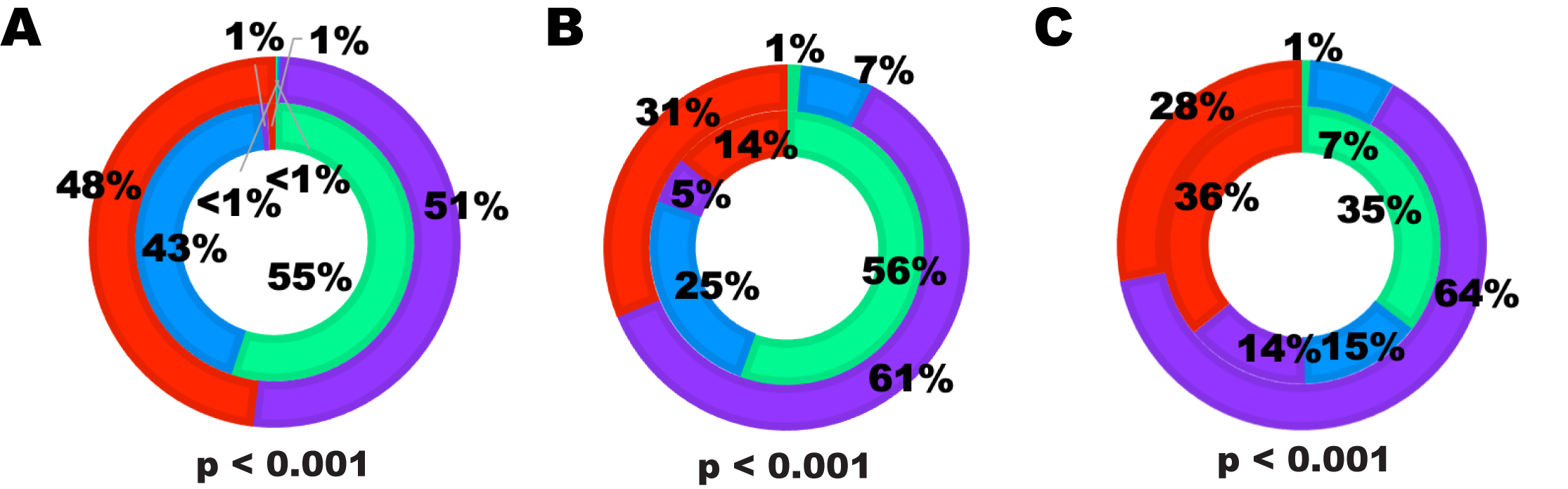

Supplement: S5 Fig — (A-C) Procyclic (A), long nectomonad (B) and leptomonad (C) stages per quadrant (inner circles) and the proportion of DE transcripts per quadrant in long nectomonad (A), leptomonad (B), and metacyclic (C) stages (outer circles). Differences were statistically significant at p < 0.0001 (Chi-square test). (TIF) [file pntd.0008014.s005.tif]
